# New Antithrombotic Drugs in Acute Coronary Syndrome

**DOI:** 10.3390/jcm9072059

**Published:** 2020-06-30

**Authors:** Bastiaan Zwart, William A. E. Parker, Robert F. Storey

**Affiliations:** 1 Department of Cardiology, St. Antonius Hospital, 3435 CM Nieuwegein, The Netherlands; 2 Department of Cardiology, Catharina Hospital, 5623 EJ Eindhoven, The Netherlands; 3 Cardiovascular Research Unit, Department of Infection, Immunity and Cardiovascular Disease, University of Sheffield, Sheffield S10 2RX, UK; w.parker@sheffield.ac.uk (W.A.E.P.); r.f.storey@sheffield.ac.uk (R.F.S.); 4 South Yorkshire Cardiothoracic Centre, Sheffield Teaching Hospitals NHS Foundation Trust, Sheffield S5 7AU, UK

**Keywords:** acute coronary syndrome, platelets, antithrombotic drugs

## Abstract

In recent years, much progress has been made in the field of antithrombotic drugs in acute coronary syndrome (ACS) treatment, as reflected by the introduction of the more potent P2Y12-inhibitors prasugrel and ticagrelor, and novel forms of concomitant anticoagulation, such as fondaparinux and bivalirudin. However, despite substantial improvements in contemporary ACS treatment, there remains residual ischemic risk in this group and hence the need for even more effective antithrombotic drugs, while balancing antithrombotic efficacy against bleeding risk. This review discusses recently introduced and currently developed antiplatelet and anticoagulant drugs in ACS treatment.

## 1. Introduction

Aspirin is the cornerstone of the antithrombotic management of coronary artery disease and other atherothrombotic diseases. Whereas venous thromboembolism (VTE) has traditionally been treated with heparins and other forms of anticoagulation, it is recognized that arterial thrombi have a different composition, with more platelets and less fibrin, and therefore need a different treatment strategy [1]. In patients with acute coronary syndrome (ACS), a combination of aspirin, heparin, and fibrinolytic agents was previously used. Despite developments in the treatment of ACS, such as improvements in percutaneous coronary interventions (PCI), recurrent ischemic events were frequent. This prompted the development of more potent platelet inhibitors. The use of glycoprotein (GP) IIb/IIIa inhibitors (GPI)—parenteral agents (e.g., abciximab, tirofiban) that reduce platelet aggregation by blocking the GPIIb/IIIa receptor on the platelet surface—was found to reduce early adverse cardiac events, including angioplasty failure and restenosis [2,3]. However, GPI treatment was complicated by high bleeding rates. Another frequent complication of PCI was vessel closure due to stent thrombosis. Ticlopidine was an orally-administered antiplatelet agent that proved to be effective in improving the patency of intracoronary stents and was subsequently shown to act by blocking the platelet P2Y_12_ receptor, a receptor for adenosine diphosphate (ADP) that plays a central role in amplifying platelet activation [4,5,6]. Ticlopidine was succeeded by clopidogrel, another P2Y_12_-inhibitor with fewer side-effects that was shown to reduce myocardial infarction, cardiovascular death, and stroke in aspirin-treated ACS patients, including those treated with PCI [7,8]. Nowadays, dual antiplatelet therapy (DAPT) consisting of aspirin with a P2Y_12_-inhibitor is the gold standard for the treatment of ACS and after PCI, whereas GPI use is mostly limited to bail-out indications in cases of no-reflow or thrombotic complications (class IIa) [9,10,11].

**Figure 1:**
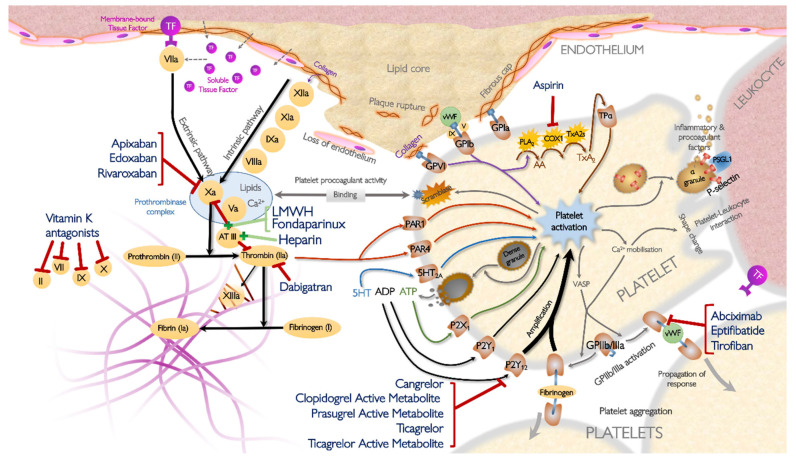
Mechanisms of action of established antithrombotic drugs in the context of the atherothrombotic response. Modified from Parker & Storey (Thrombotic Response, ESC Textbook of Cardiovascular Medicine 3rd edition, 2018, Oxford University Press). 5HT, 5-hydroxytryptamine (serotonin); AA, arachidonic acid; ADP, adenosine diphosphate; ATIII, antithrombin III; ATP, adenosine triphosphate; Ca^2+^, calcium; COX1, cyclo-oxygenase 1; GP, glycoprotein; IXa, activated factor IX; P2 × 1, platelet ATP receptor; LMWH, low- molecular weight heparin; P2Y_1_/P2Y_12_, platelet ADP receptors; PAR, protease activated receptor; PLA2, phospholipase A2; PSGL1, P-selectin glycoprotein ligand 1; TF, tissue factor; TPα, thromboxane receptor α; TXA2, thromboxane A2; TXA2s, thromboxane A2 synthase; Va, activated factor V; VIIa, activated factor VII; VIIIa, activated factor VIII; VASP, vasodilator-stimulated phosphoprotein; vWF, von Willebrand factor; Xa, activated factor X; XIa, activated factor XI; XIIa, activated factor XII; XIIIa, activated factor XII.

**Figure 2:**
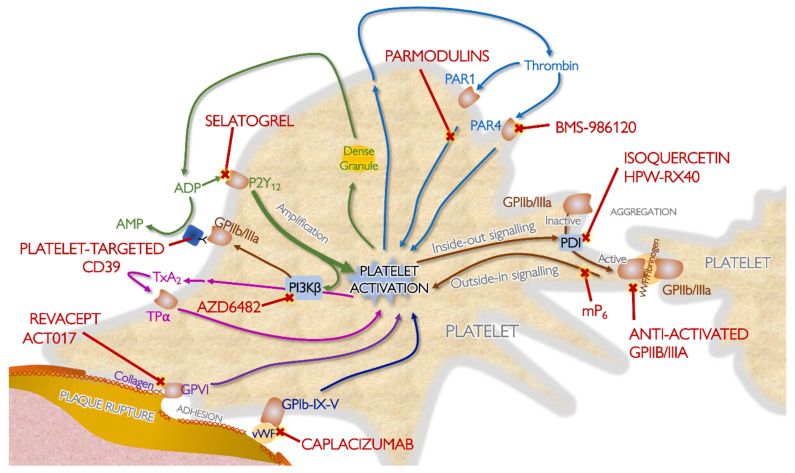
Examples of novel antiplatelet agents and their mechanisms of action. ADP, adenosine diphosphate; AMP, adenosine monophosphate; CD, cluster of differentiation; GP, glycoprotein; P2Y_12_, platelet ADP receptor; PAR, protease activated receptor; PDI, protein disulphide isomerase; PI3Kbeta, phosphoinositide 3-kinase; TPa, thromboxane receptor a; TxA2, thromboxane A2; vWF, von Willebrand factor.

In recent years, much further progress has been made in the field of antithrombotic drugs in ACS, as reflected by the introduction of the more potent P2Y_12_-inhibitors prasugrel and ticagrelor [12,13]. Novel forms of concomitant anticoagulation, which is now routinely added in ACS [9,10], have been developed, such as fondaparinux, a pentasaccharide with indirect anti-factor (F)Xa activity, and bivalirudin, a direct thrombin inhibitor. However, despite substantial improvements in contemporary ACS treatment, there remains residual ischemic risk in this group and hence the need for even more effective antithrombotic drugs. On the other hand, patients treated with potent antithrombotic drugs are exposed to a substantial bleeding risk. Therefore, the ultimate goal is to develop effective antithrombotic drugs with minimal hemorrhagic complications. This review discusses recently introduced and currently developed antiplatelet and anticoagulant drugs in ACS. The drugs discussed in this review and their mechanisms of action are summarized in Table 1 and Figure 1 and Figure 2.

## 2. Cangrelor

As described, DAPT is now the cornerstone of ACS treatment. However, currently used oral P2Y_12_-inhibitors have their limitations. Clopidogrel is a prodrug that needs to be metabolized by hepatic cytochrome P450 (CYP) enzymes into its active form and therefore has a delayed onset of action. The more potent P2Y_12_-inhibitors prasugrel and ticagrelor, which are used in contemporary ACS treatment, have a faster onset of action as compared to clopidogrel and achieve high levels of platelet inhibition within 1–2 h in stable patients [14,15]. However, none of the currently available oral P2Y_12_-inhibitors achieve instant antiplatelet inhibition, which is desirable especially when patients undergo PCI. When opioids are administered, which is common in the ST-elevation myocardial infarction (STEMI) setting, absorption of prasugrel or ticagrelor is known to be even more delayed, which exposes these patients to an increased risk of stent thrombosis, a dangerous complication of coronary stenting [16,17,18,19]. Parenteral administration of GPI may be considered as an alternative strategy to mitigate the risk of acute stent thrombosis in these patients [20], but the use of GPI bears a considerable bleeding risk and thus might not be an attractive strategy in all patients [21]. Another limitation of all oral P2Y_12_-inhibitors is the delayed recovery of platelet reactivity after discontinuation, which is relevant when anticipating urgent surgery. Recovery of platelet function following cessation varies from 3–7 days depending on the type of P2Y_12_-inhibitor and, in the case of clopidogrel, the level of platelet inhibition achieved during treatment [22,23].

Cangrelor, an intravenous, reversibly-binding platelet P2Y_12_ receptor antagonist, has the potential to overcome the above-mentioned limitations of the oral P2Y_12_-inhibitors. It has an ultra-quick onset and offset of action and therefore appears very suitable for PCI in the acute STEMI setting. Due to the intravenous method of administration, cangrelor has high bioavailability and is highly potent, with near-complete inhibition of ADP-induced platelet aggregation within 2 min after bolus injection, following which its therapeutic effects are maintained throughout infusion [24,25]. After discontinuation of cangrelor, platelet reactivity recovers back to normal within 1–2 h, due to a mean plasma half-life of 5–10 min.

Efficacy of cangrelor in clinical practice was assessed in the Cangrelor versus standard therapy to achieve optimal management of platelet inhibition (CHAMPION) trials. The CHAMPION-PCI, CHAMPION-PLATFORM, and CHAMPION-PHOENIX were phase III trials that compared cangrelor to either clopidogrel or placebo [26,27,28]. In an individual patient-level meta-analysis of these three trials containing 24,910 patients, cangrelor was found to be effective in reducing the primary outcome of death, myocardial infarction and ischemia-driven revascularization as well as stent thrombosis [29]. In this same meta-analysis, an increase in GUSTO mild bleeding was observed, but not GUSTO moderate bleeding or transfusion rates.

Current European Society of Cardiology (ESC) guidelines indicate that cangrelor may be used in P2Y_12_-inhibitor naïve patients undergoing PCI with stable coronary artery disease, non-ST-elevation-ACS (NSTE-ACS), or STEMI [9,10,11]. Of note, the benefit of adding cangrelor to the faster acting P2Y_12_-inhibitors ticagrelor and prasugrel remains unclear, as no phase III trials compared cangrelor to these agents [30]. A further caveat is that cangrelor blocks the binding of thienopyridine active metabolites to the P2Y_12_ receptor and so the administration of loading doses of prasugrel or clopidogrel should be deferred until the end of the cangrelor infusion [31].

In conclusion, cangrelor provides fast-onset and rapidly reversible platelet inhibition and is effective and safe to use in PCI compared to clopidogrel or placebo. It is approved in patients undergoing PCI who have not been pre-loaded with a P2Y_12_-inhibitor and are not being treated with GPI [32]. In clinical practice, indications might include high-risk patients who undergo PCI without proper pre-loading with an oral P2Y_12_-inhibitor (e.g., patients undergoing ad hoc high-risk PCI or STEMI patients presenting with resuscitated cardiac arrest) or opiate-treated STEMI patients in whom absorption of oral P2Y_12_ inhibitors is known to be delayed [33]. Furthermore, cangrelor might be used as a ‘bridging’ option in patients with recent PCI who need to undergo surgery after discontinuation of oral platelet therapy [22]. Further clinical studies and registries are needed to investigate the use of cangrelor in conjunction with ticagrelor or prasugrel.

## 3. Novel Antiplatelet Drugs

Several novel antiplatelet drugs have recently been developed. Some exploit novel targets whilst others seek to refine existing drugs in a class. None have yet reached phase III studies in patients with ACS, but clearly there are signs of potential for future use should they be subject to rigorous clinical testing, including in this population to establish safety and efficacy.

### 3.1. Selatogrel

Selatogrel is a novel, parenterally-active, reversibly-binding P2Y_12_ inhibitor formulated for subcutaneous (SC) administration [34,35]. Its molecular structure is derived from incorporating the pyrimidine group of ticagrelor into a family of compounds previously investigated as P2Y_12_ receptor antagonists [36]. Preclinical studies suggested that selatogrel was potent and selective, but also that it might have a wider therapeutic index when compared to clopidogrel or ticagrelor with regards to increase in bleeding risk whilst maintaining antithrombotic effect [37]. Phase I studies of oral selatogrel or prodrug were hindered by poor absorption and palatability [38]. Subsequently, the SC preparation of selatogrel was tested and its safety and tolerability demonstrated [39]. The drug has rapid onset and a radiolabeled drug study suggested no significant plasma metabolites and that elimination was largely faecal, predicting no significant drug–drug interaction [40]. Phase II studies in both acute and chronic settings of ischemic heart disease have now been reported with promising results. In the largest, 345 patients receiving maintenance antiplatelet therapy for chronic coronary syndromes were randomized to receive SC selatogrel, at one of two doses, or placebo [41]. Selatogrel reliably and potently inhibited platelet reactivity by 30 min for around 8 h, the effect wearing off by 24 h. Importantly, selatogrel’s effect appeared additive even in those already receiving oral P2Y_12_ inhibitors, and there were no incidences of major bleeding. The drug’s profile of effect was broadly similar when tested in 47 patients with acute MI [42]. The clinical setting(s) in which selatogrel may find a niche remains to be determined but, given it provides potent, rapid, and reversible P2Y_12_ inhibition without the need for intravenous access nor an infusion, it provides a promising option for early pre-hospital administration by caregivers or even self-administration by patients during a suspected ACS event, circumventing the issue of delayed absorption of oral P2Y_12_ inhibitors by opioids [16]. Consequently, selatogrel might provide benefits over existing standard care and warrants further study in phase III trials.

### 3.2. Phosphoinositide 3-Kinase β

The enzyme phosphoinositide 3-kinase (PI3K) plays a central role in many cellular signaling systems through the activation of Akt (also known as protein kinase B), and this includes within platelets [43]. The PI3K-1A subtype, including p110α, p110β or p110δ catalytic subunits, is crucial to cell signaling during platelet activation by collagen. Moreover, the p110β subunit, the presence of which designates the enzyme PI3Kβ, has particularly important roles in, for example, the generation of thromboxane A_2_ upon ADP stimulation and in sustaining function of glycoprotein (GP) IIb/IIIa (also known as integrin α_IIb_β_3_), responsible for the common final pathway of platelet aggregation [44]. PI3Kβ therefore represents a rational target for antiplatelet therapy, made more attractive by the fact that in animal studies its inhibition appears to insignificantly affect primary hemostatic mechanisms [45].

Several PI3Kβ inhibitors have been developed, but human studies have been limited to date. AZD6482 is a selective, potent, and ATP-competitive inhibitor of PI3Kβ that is the active enantiomer of a previously developed racemic mixture [46]. In a study of 40 male healthy volunteers randomized to receive escalating intravenous doses of AZD6482 or placebo, the drug mildly inhibited platelet aggregation responses assessed using a number of methods and agonists. Whilst bleeding time correlated positively with plasma drug levels, the strength of the effect was judged to be small. PI3Kβ also appears to play an important role in tumor progression and another PI3Kβ inhibitor, orally-active GSK2636771, has been tested in a first-in-man study of 65 patients with advanced solid organ malignancy [47]. However, effects on platelet function were not evaluated.

### 3.3. GP IIb/IIIa Outside-In Signaling

GP IIb/IIIa is of importance in the final common pathway of platelet aggregation. Once activated by intracellular processes such as vasodilator-stimulated phosphoprotein dephosphorylation and calcium mobilization, surface GP IIb/IIIa forms crosslinks with GP IIb/IIIa on other platelets via fibrinogen or von Willebrand factor (vWF) bridges [48]. This can be prevented by the currently-used GP IIb/IIIa inhibitors such as tirofiban, abciximab, and eptifibatide [49]. However, GP IIb/IIIa not only acts as an effector of platelet aggregation through inside-out signaling but also has a role as a receptor in outside-in signaling via numerous pathways leading to amplification of platelet activation, thus propagating thrombosis [50]. Inhibition of outside-in signaling, whilst maintaining inside-out signaling, has been hypothesized to offer the attractive combination of preserving the integrity of the primary hemostatic response whilst preventing the propagation of platelet aggregation that leads to vessel occlusion during thrombosis [51]. Targeting the interaction of Gα_13_ and the IIIa subunit may achieve this. A peptide, mP_6_, incorporating the amino acid ExE motif, has been developed as a putative drug, and in animal studies it inhibited the second wave of platelet activation and the propagation of thrombosis in an arterial injury model, without prolonging bleeding time [51]. No human studies have yet been reported.

### 3.4. Conformation-Specific Targeting of GP IIb/IIIa

Another strategy that has been explored for more targeted inhibition of GP IIb/IIIa only on activated platelets is based on exploiting the conformational change that occurs upon activation of GP IIb/IIIa, exposing the ligand-binding pocket of the receptor. A single chain antibody that binds to GP IIb/IIIa in its active conformation only has been developed and tested in animal models, in which it inhibited thrombus propagation without significant effect on the bleeding time [52].

### 3.5. Activated Platelet-Targeted CD39 Therapy

Building on the idea of targeting activated platelets via conformation-dependent binding of a single chain antibody to GP IIb/IIIa, a further strategy has been developed and tested in preclinical studies. This involves linking the single chain antibody to the naturally-occuring ecto-nucleoside triphosphate diphosphohydrolase CD39, which therefore degrades adenosine diphosphate (ADP) in the locality of the cell membrane of the activated platelet, thus preventing ADP from stimulating platelets via P2Y_1_ and, most importantly, P2Y_12_ receptors [53]. In human platelet-rich plasma, the antibody–CD39 complex was more effective at inhibiting platelet aggregation in response to ADP stimulation than adding non-targeted CD39 and, in animal models, it prevented occlusive thrombus without prolongation of bleeding time [53].

### 3.6. Inhibitors of Platelet GP VI

Platelets are exposed to large amounts of collagen during atherothrombotic events, and collagen is a key agonist in the initiation and propagation of thrombosis, including through stimulation of platelet GP VI receptors [54]. Presently, no clinically approved drugs directly target platelet collagen receptors. Recently, however, attention has turned to targeting GP VI. Revacept (soluble dimeric glycoprotein VI-Fc fusion protein) has been developed as an inhibitor. In a phase I study of 30 healthy volunteers who received a single administration of intravenous revacept, the drug dose-dependently inhibited collagen-induced platelet aggregation for up to seven days and appeared safe and well-tolerated [55]. A phase II double-blind randomized controlled trial (dbRCT) of revacept vs. placebo in 158 patients with symptomatic carotid artery stenosis, transient ischemic attacks, amaurosis fugax, or stroke was completed in 2018 (NCT01645306) but the full results have not yet been made available. A second placebo-controlled dbRCT phase II study of 334 patients undergoing elective PCI, Intracoronary Stenting and Antithrombotic Regimen: Lesion Platelet Adhesion as Selective Target of Endovenous Revacept (ISAR-PLASTER), has also recently finished and results are awaited [56].

Similarly, a humanized antigen-binding fragment (Fab) against GP VI, ACT017, has been formulated [57]. This too has now been tested in a phase I study of healthy volunteers, who were randomized to receive varying doses of intravenous ACT017 or placebo. Again, the drug was well-tolerated, appeared safe, and dose-dependently inhibited collagen-induced platelet aggregation [58].

### 3.7. Inhibition of Protein Disulfide Isomerase

The enzyme protein disulfide isomerase (PDI) has a role in the conformational changes associated with the activation of GP IIb/IIIa [59]. Extracellular PDI also promotes thrombin generation by activation of factor V, thrombin being a potent activator of both cellular and acellular thrombosis. Inhibition of PDI attenuates these processes without significantly affecting bleeding time [60].

Isoquercetin is a flavonoid that inhibits PDI and a phase II study has now been reported in the setting of prevention of cancer-associated thrombosis, with pharmacodynamic effects seen, such as reduction in circulating levels of D-dimer and platelet-dependent thrombin generation [61]. A further small phase II/III study is ongoing in this population (NCT02195232), but the effects of isoquercetin have not been studied in those with atherothrombotic cardiovascular disease.

Another PDI inhibitor, HPW-RX40, a derivative of β-nitrostyrene, has also been shown to reduce thrombus formation in vitro and in animal studies [62].

### 3.8. Inhibition of Protease-Activated Receptors

Thrombin, generated both by activation of the coagulation cascade and by platelets via surface cell membrane scramblase activity, stimulates platelets via protease-activated receptor (PAR) 1 and, at high levels, PAR4 [63]. Previously, PAR1 inhibitors such as vorapaxar showed great promise as an antiplatelet agent that in preclinical studies provided potent effect without prolonging bleeding time, and the drug was tested in two large double blind randomized controlled trials of patients with atherothrombotic cardiovascular disease [64,65]. Although vorapaxar reduced ischemic events and became licensed for clinical use, there was a disappointing excess of bleeding risk that has limited its widespread adoption. It has been suggested that counterproductive inhibition of PAR1-related endothelial cytoprotective signaling pathways resulting in endothelial injury and potentially loss of vessel wall integrity that might contribute to the increased bleeding risk [66]. A new class of PAR1 inhibitor, the parmodulins, have been developed with the intention of inhibiting selectively PAR1-dependent pathways relevant to thrombosis but avoiding any anti-cytoprotective effects (34). Preclinical studies have been encouraging, but clinical studies would clearly be needed to determine if parmodulins have a less detrimental effect on bleeding risk than vorapaxar.

PAR4 has recently been considered as an alternative target to reduce thrombin-induced platelet activation. A novel PAR4 inhibitor, BMS-986120, demonstrated reduced thrombus formation without prolongation of the bleeding time in monkeys [67]. In a subsequent phase I study of 40 healthy volunteers, a single oral dose of BMS-986120 selectively and reversibly inhibited ex vivo platelet-rich thrombus formation upon stimulation with PAR4 agonist peptide in high-shear-stress conditions [68]. In contrast to aspirin or clopidogrel, BMS-986120 had no effect in ex vivo low-shear-stress conditions, which may be promising for bleeding risk although of course requires in vivo evaluation.

### 3.9. Caplacizumab

A novel antiplatelet agent has recently been licensed for the treatment of adults with acquired thrombotic thrombocytopenic purpura (aTTP), when used in combination with plasma exchange and immunosuppressive drugs. Caplacizumab is a humanized, bivalent, variable domain immunoglobulin fragment, which targets the A1 domain of von Willebrand factor, inhibiting interaction with the platelet glycoprotein Ib-IX-V receptor, which has an important role in platelet adhesion to damaged sub-endothelium and thus is also a potential target for antiplatelet therapy in atherothrombosis. A single intravenous dose of caplacizumab significantly inhibits platelet adhesion, as measured with ristocetin assays, for at least 24 h, and subsequent maintenance subcutaneous administration continues to exert this effect [69]. Whilst efficacy has been proven for treatment of aTTP in a modestly sized phase III study [70], the drug has not yet been investigated in other thrombotic conditions. Capcizumab led to significantly greater risk of gingival bleeding and epistaxis when compared to placebo.

## 4. Anticoagulation in ACS

In both STEMI and NSTE-ACS, parenteral anticoagulation is recommended [10,11] in addition to antiplatelet therapy, and should be administered at the time of diagnosis. It reduces the generation and/or action of thrombin and thereby targets another pathway in thrombus generation [71]. Several parenteral options are available, which are usually only used as a short course. In NSTE-ACS, the selective FXa inhibitor fondaparinux is the anticoagulant of choice in patients who do not proceed directly to coronary angiography, based on its favorable efficacy–safety profile [10,72]. In STEMI patients and in NSTE-ACS patients who have not been pretreated with anticoagulation, unfractionated heparin (UFH) is the most common anticoagulant used during PCI.

### 4.1. Enoxaparin in STEMI

Current ESC guidelines advise that enoxaparin should be considered as an alternative anti-coagulant agent to UFH [9,11]. Enoxaparin is a low-molecular-weight heparin (LMWH) that inhibits FXa and, to a lesser extent, factor IIa (thrombin) [73]. From a mechanistic point of view, enoxaparin might be more effective than UFH by targeting a more proximal part of the coagulation cascade. It provides a more predictable antithrombotic effect compared with UFH and therefore does not require monitoring [74]. Furthermore, it has the ability to provide inhibition of thrombin-induced platelet activation [75]. Evidence of a beneficial effect in STEMI stems primarily from the ATOLL trial [76] in which STEMI patients undergoing primary PCI were randomized to either enoxaparin or UFH. A reduction of the main secondary end point, comprising a composite of death, recurrent ACS, or urgent revascularization, was observed with enoxaparin. However, the results of this study might no longer be applicable to current practice, as this study was performed before the introduction of ticagrelor (almost all patients in the study were treated with clopidogrel) and a high proportion of patients received concomitant GPI therapy. Recently, the PENNY PCI study was published [75], investigating the pharmacodynamic effects of enoxaparin regimen in primary PCI in contemporary practice. Enoxaparin was administered in a dose of 0.75 mg/kg (bolus) followed by infusion of enoxaparin 0.75 mg/kg/6 h. Anti-FXa levels were measured at four time points before, during and after PCI. Enoxaparin was found to result in sustained anti-Xa levels during infusion and no bleeding complications were observed. Therefore, enoxaparin might be an attractive alternative to UFH in primary PCI. In particular, it might be an alternative to cangrelor or GPI for addressing the delayed absorption of oral P2Y_12_-inhibitors, especially in morphine-treated STEMI patients undergoing PCI [17].

### 4.2. Non-Vitamin-K-Antagonist Oral Anticoagulants (NOACs)

The combination of DAPT with oral anticoagulation is known to bear a two- to three-fold higher bleeding risk without an apparent benefit in terms of ischemic risk in patients with atrial fibrillation undergoing PCI or with ACS [77,78]. However, the introduction of the safer NOACs raised the question of whether there might yet be a window of benefit of adding NOAC to DAPT in ACS patients without a formal indication for oral anticoagulation. To date, several studies have investigated such a strategy.

Dabigatran on top of DAPT was evaluated in the RE-DEEM study, which was a phase II study that included 1861 NSTE-ACS and STEMI patients. Various doses, from 50 mg b.d. to 150 mg b.d., were evaluated against placebo and a dose-dependent increase in major or clinically-relevant minor bleeding was found in the NOAC groups without significant differences in ischemic outcomes [79]. The APPRAISE-2 trial compared apixaban with placebo in 7392 ACS patients with two additional high-risk features. Patients were treated with either aspirin monotherapy (16.3%) or aspirin and clopidogrel (79%) and were randomized to receive concomitant apixaban in a dose of 5 mg b.d. (or 2.5 mg b.d. in case of a creatinine clearance < 40 mL/min) or placebo [80]. The addition of apixaban did not significantly reduce the composite ischemic end point but led to an excess of Thrombolysis in Myocardial Infarction (TIMI) major bleeding (HR 2.59), including intracranial and fatal bleeding. Consequently, the study was stopped prematurely. Recently, the AUGUSTUS trial, which had a 2 × 2 factorial design comparing NOAC vs. VKA and aspirin vs. placebo in patients with atrial fibrillation and either ACS or undergoing PCI (or both), confirmed that dual therapy with full-dose apixaban and an oral P2Y_12_ inhibitor (i.e., with the omittance of aspirin) is safer than triple therapy, again showing no significant differences in ischemic outcomes although the study was underpowered for this endpoint [81].

Rivaroxaban, another direct FXa inhibitor, has been studied in this setting in the ATLAS ACS– TIMI phase II trial and subsequently in the ATLAS ACS 2–TIMI 51 phase III trial [82,83]. This large-scale study included 15,526 patients with recent ACS (within seven days after admission). All patients received standard medical treatment and were randomized in a 1:1:1 fashion to a regimen of either 2.5 mg b.d. or 5 mg b.d. of rivaroxaban or placebo. Importantly, in this study, a reduced-dose NOAC was used as compared to the “full dose” rivaroxaban 20 mg o.d. The addition of rivaroxaban reduced the composite endpoint of death from cardiovascular causes, myocardial infarction, or stroke (8.9% vs. 10.7%, *p* = 0.008), although this benefit was counterbalanced by higher rates of both major bleeding unrelated to coronary-artery bypass grafting (2.1% vs. 0.6%, *p* < 0.001) and intracranial hemorrhage (0.6% vs. 0.2%, *p* = 0.009). However, a reduction in rates of death from both cardiovascular causes (2.7% vs. 4.1%, *p* = 0.002) and from any cause (2.9% vs. 4.5%, *p* = 0.002) was observed in the rivaroxaban 2.5 mg b.d. group.

Finally, several other emerging NOACs have been investigated in the ACS setting but no positive results were observed in these phase II trials [84,85].

In conclusion, so far only rivaroxaban has been shown to reduce ischemic events and mortality in patients with ACS when a reduced dose of 2.5 mg b.d. was used in conjunction with standard ACS treatment. However, rivaroxaban was not studied in the setting of the more potent P2Y_12_-inhibitors prasugrel and ticagrelor. Current ESC guidelines indicate that low-dose rivaroxaban 2.5 mg b.d. “may be considered” (IIb) if ischemic risk exceeds bleeding risk in patients treated with aspirin and clopidogrel.

### 4.3. Development of Factor IX, XI, and XII Inhibitors

Looking to the horizon of anticoagulant therapy, the development of FIX, FXI, and FXII inhibitors seems promising. In the last decade, much research has focused on these specific coagulation factors. FXI is the first protein in the hemostatic pathway of intrinsic blood coagulation. FXI activates FIX, whereas factor XI itself is activated by Factor XII (FXII), a component of the contact system together with the proteins prekalikrein and H-kininogen [86]. Attempts to target these upstream factors arose from the observation that patients deficient in FXI or FXII suffer no increase or only mild increase in bleeding events, respectively [87]. On the other hand, in vivo research showed that FXI- or FXII-deficient animals seem to be protected from thrombotic complications [88,89,90]. Similarly, molecular genetic studies in FIX-deficient mice showed a correlation between in vivo FIXa activity and susceptibility to occlusive venous thrombus formation [91]. In humans, elevated levels of FIX, FXI, or FXII are all associated with prothrombotic phenotypes [92].

Following these observations, the old paradigm that thrombosis and bleeding are two sides of the same coin was challenged [93]. Is it possible to develop an antithrombotic drug without any bleeding complications? Several phase I and phase II trials are currently being conducted [94]. Whereas the first results of FIX inhibitors were not as promising as hoped [90,95], several other trials focus on FXI and FXII. FXI might be the most promising target of the two, as there is more epidemiological evidence for its role in thrombosis. Targets for the newly-developed FXI inhibitors include synthesis of FXI in the liver whereas other drugs bind FXI or FXIa, or block its active site [90,96]. Also, monoclonal antibodies are being developed. Although promising, these drugs are only currently in phase II development. Most studies focus on venous thromboembolism and whether these drugs would be effective in ACS is a further step in the future. In conclusion, FIX, FXII, and FXI have emerged as promising targets for novel anticoagulant drugs, with the potential of reducing thrombus formation with minimal effect on hemostatic pathways (i.e., bleeding). Their application in clinical practice, and in ACS in particular, is yet to be determined and further results of clinical studies are awaited.

## 5. Conclusions

Much progress has been made in the field of antithrombotic drugs in ACS in recent years. Newly introduced drugs in clinical practice are cangrelor, an intravenous P2Y_12_-antagonist, and the use of enoxaparin in STEMI and rivaroxaban as an adjunctive in ACS. Other potentially interesting drugs are currently being developed, which include several novel potent antiplatelet drugs targeting alternative pathways. Furthermore, the development of FIX, FXI, and FXII inhibitors seems promising, with the potential of reducing thrombus formation with only minimal effect on bleeding. Hence, there is a glance of several promising new antithrombotic drugs on the horizon. Their efficacy and applicability in the ACS setting needs to be further proven in clinical trials.

## Figures and Tables

**Table 1:** Of novel antithrombotic drugs tested in clinical trials.

Type of Drugs	Class of Drugs	Drugs Name(s)	Route of Administration	Mechanism of Action	Main Study Findings	Stage of Development
Anticoagulant drugs	Low-molecular-weight heparin	Enoxaparin	Subcutaneous and intravenous	inhibiting factor FXa and FIIa	Sustained anti-Xa levels during infusion in STEMI patients undergoing PCI	Launched
	Non-vitamin-K-antagonist oral anticoagulants	Rivaroxaban	Oral	direct FXa inhibitor	Addition of low-dose rivaroxaban (2.5 mg b.d.) reduced ischemic events and all-cause mortality with an increase in bleeding. No positive study results of other NOACs tested.	Launched
	Inhibitors of intrinsic pathway of coagulation	n/a	Intravenous, subcutaneous, and oral	Inhibitors of “upstream” anticoagulation factors FIX-, FXI-, and FXII. Various targets of action (e.g., hepatic synthesis, monoclonal antibodies)	Various phase I and phase II trials currently ongoing. First study results of FXI- and FXII-inhibitors more promising than FIX-inhibitors.	Phase I/II
Antiplatelet drugs	P2Y_12_-receptor antagonist	Cangrelor	Intravenous	Adenosine triphosphate analogue blocking P2Y_12_-receptor	Phase III trials show reduced MACE and stent thrombosis versus oral clopidogrel.	Launched
	P2Y_12_-receptor antagonist	Selatogrel	Subcutaneous	P2Y_12_ receptor antagonist	Potent platelet P2Y_12_ inhibition within 30 min, reversible by 24 h. No major bleeding events in the largest clinical study.	Phase II
	PI3Kβ-inhibitors	AZD6482; GSK2636771	Intravenous and oral	Inhibiting the effect of PI3Kβ which acts through platelet cellular signaling systems	Mild effect on platelet activity, minimal effect on bleeding times in healthy volunteers. GSK2636771 has been evaluated in a phase I trial for its effect on tumor progression.	Phase I
	Platelet GP VI-inhibitors	Revacept; ACT017	Intravenous	Inhibition of collagen-induced platelet aggregation	In phase I studies, drugs appeared to be effective and safe. Two phase II studies completed but results have not been fully disclosed yet.	Phase II
	Protein disulfide isomerase (PDI) inhibitors	Isoquercetin HPW-RX40	Oral	Inhibition of PDI attenuates conformational changes in the activation of GP IIb/IIIa and inhibits the generation of thrombin generation	In the setting of prevention of cancer-associated thrombosis, isoquercetin caused a reduction in circulating levels of D-dimer and platelet-dependent thrombin generation was demonstrated. HPW-RX40 has only been tested in preclinical studies.	Phase II
	PAR1 signaling modulators	Parmodulins	n/a	Inhibition of PAR1 signaling pathways involved in platelet activation, but not those relevant to endothelial cytoprotective effects	Preclinical studies have demonstrated inhibition of thrombin-induced platelet activation.	Preclinical
	PAR4-inhibitors	BMS-986120	Oral	Inhibition of PAR4 activation by thrombin	In a phase I study of healthy volunteers, BMS-986120 inhibited ex vivo platelet-rich thrombus formation upon stimulation with PAR4 agonist peptide in high-shear-stress conditions.	Phase I
	Platelet glycoprotein Ib-IX-V receptor inhibitor	Caplacizumab	Intravenous	Caplacizumab is a immunoglobulin fragment, which targets the A1 domain of von Willebrand factor, inhibiting interaction with the platelet glycoprotein Ib-IX-V receptor, which has an important role in platelet adhesion to damaged sub-endothelium.	Efficacy has been proven in a modestly sized phase III study for treatment of aTTP with an increase in gingival bleeding and epistaxis. No studies have yet been performed in other thrombotic conditions.	Launched (for aTTP)
	Confirmation-specific GPIIb/IIIa inhibitors	Anti-activated GPIIb/IIIa	n/a	Activated, but not unactivated GPIIb/IIIa is inhibited	Inhibited propagation of thrombosis in an animal model without prolonging bleeding time.	Preclinical
	Inhibitors of GPIIb/IIIa outside-in signaling	mP_6_	n/a	Disrupts interaction between Gα_13_ and IIIa, inhibiting downstream signaling	Inhibited propagation of thrombosis in an animal model without prolonging bleeding time.	Preclinical
	Platelet-targeted CD39	CD39-anti GPIIb/IIIa	n/a	CD39 breaks down ADP. Linking CD39 to anti-GPIIb/IIIa targets the enzyme to platelets.	Preclinical studies have shown greater antiplatelet efficacy of platelet-targeted CD39 compared to untargeted CD39.	Preclinical

## References

[1] Handin R.I. (1996). Platelets and coronary artery disease. N. Engl. J. Med..

[2] EPIC Investigators (1994). Use of a monoclonal antibody directed against the platelet glycoprotein IIb/IIIa receptor in high-risk coronary angioplasty. N. Engl. J. Med..

[3] Restore Investigators (1997). Effects of platelet glycoprotein IIb/IIIa blockade with tirofiban on adverse cardiac events in patients with unstable angina or acute myocardial infarction undergoing coronary angioplasty. The RESTORE Investigators. Randomized Efficacy Study of Tirofiban for Outcomes and REstenosis. Circulation.

[4] Schömig A., Neumann F.J., Kastrati A., Schühlen H., Blasini R., Hadamitzky M., Walter H., Zitzmann-Roth E.M., Richardt G., Alt E. (1996). A randomized comparison of antiplatelet and anticoagulant therapy after the placement of coronary-artery stents. N. Engl. J. Med..

[5] Leon M.B., Baim D.S., Popma J.J., Gordon P.C., Cutlip D.E., Ho K.K., Giambartolomei A., Diver D.J., Lasorda D.M., Williams D.O. (1998). A clinical trial comparing three antithrombotic-drug regimens after coronary-artery stenting. Stent Anticoagulation Restenosis Study Investigators. N. Engl. J. Med..

[6] Patrono C., Morais J., Baigent C., Collet J.-P., Fitzgerald D., Halvorsen S., Rocca B., Siegbahn A., Storey R.F., Vilahur G. (2017). Antiplatelet Agents for the Treatment and Prevention of Coronary Atherothrombosis. J. Am. Coll. Cardiol..

[7] Yusuf S., Zhao F., Mehta S.R., Chrolavicius S., Tognoni G., Fox K.K. (2001). Clopidogrel in Unstable Angina to Prevent Recurrent Events Trial Investigators Effects of clopidogrel in addition to aspirin in patients with acute coronary syndromes without ST-segment elevation. N. Engl. J. Med..

[8] Mehta S.R., Yusuf S., Peters R.J., Bertrand M.E., Lewis B.S., Natarajan M.K., Malmberg K., Rupprecht H., Zhao F., Chrolavicius S. (2001). Effects of pretreatment with clopidogrel and aspirin followed by long-term therapy in patients undergoing percutaneous coronary intervention: The PCI-CURE study. Lancet.

[9] Neumann F.J., Sousa-Uva M., Ahlsson A., Alfonso F., Banning A.P., Benedetto U., Byrne R.A., Collet J.P., Falk V., Head S.J. (2019). 2018 ESC/EACTS Guidelines on myocardial revascularization. Eur. Heart J..

[10] Roffi M., Patrono C., Collet J.P., Mueller C., Valgimigli M., Andreotti F., Bax J.J., Borger M.A., Brotons C., Chew D.P. (2016). 2015 ESC Guidelines for the management of acute coronary syndromes in patients presenting without persistent ST-segment elevation: Task Force for the Management of Acute Coronary Syndromes in Patients Presenting without Persistent ST-Segment Elevation of the European Society of Cardiology (ESC). Eur. Heart J..

[11] Ibanez B., James S., Agewall S., Antunes M.J., Bucciarelli-Ducci C., Bueno H., Caforio A.L.P., Crea F., Goudevenos J.A., Halvorsen S. (2017). 2017 ESC Guidelines for the management of acute myocardial infarction in patients presenting with ST-segment elevation: The Task Force for the management of acute myocardial infarction in patients presenting with ST-segment elevation of the European Society of Cardiology (ESC). Eur. Heart J..

[12] Wiviott S.D., Braunwald E., McCabe C.H., Montalescot G., Ruzyllo W., Gottlieb S., Neumann F.-J., Ardissino D., De Servi S., Murphy S.A. (2007). Prasugrel versus clopidogrel in patients with acute coronary syndromes. N. Engl. J. Med..

[13] Wallentin L., Becker R.C., Budaj A., Cannon C.P., Emanuelsson H., Held C., Horrow J., Husted S., James S., Katus H. (2009). Ticagrelor versus clopidogrel in patients with acute coronary syndromes. N. Engl. J. Med..

[14] Gurbel P.A., Bliden K.P., Butler K., Tantry U.S., Gesheff T., Wei C., Teng R., Antonino M.J., Patil S.B., Karunakaran A. (2009). Randomized double-blind assessment of the ONSET and OFFSET of the antiplatelet effects of ticagrelor versus clopidogrel in patients with stable coronary artery disease: The ONSET/OFFSET study. Circulation.

[15] Wallentin L., Varenhorst C., James S., Erlinge D., Braun O.O., Jakubowski J.A., Sugidachi A., Winters K.J., Siegbahn A. (2008). Prasugrel achieves greater and faster P2Y12receptor-mediated platelet inhibition than clopidogrel due to more efficient generation of its active metabolite in aspirin-treated patients with coronary artery disease. Eur. Heart J..

[16] Thomas M.R., Morton A.C., Hossain R., Chen B., Luo L., Shahari N.N.B.M., Hua P., Beniston R.G., Judge H.M., Storey R.F. (2016). Morphine delays the onset of action of prasugrel in patients with prior history of ST-elevation myocardial infarction. Thromb. Haemost..

[17] Kubica J., Adamski P., Ostrowska M., Sikora J., Kubica J.M., Sroka W.D., Stankowska K., Buszko K., Navarese E.P., Jilma B. (2016). Morphine delays and attenuates ticagrelor exposure and action in patients with myocardial infarction: The randomized, double-blind, placebo-controlled IMPRESSION trial. Eur. Heart J..

[18] Parodi G., Bellandi B., Xanthopoulou I., Capranzano P., Capodanno D., Valenti R., Stavrou K., Migliorini A., Antoniucci D., Tamburino C. (2015). Morphine is associated with a delayed activity of oral antiplatelet agents in patients with ST-elevation acute myocardial infarction undergoing primary percutaneous coronary intervention. Circ. Cardiovasc. Interv..

[19] Silvain J., Storey R.F., Cayla G., Esteve J.-B., Dillinger J.-G., Rousseau H., Tsatsaris A., Baradat C., Salhi N., Hamm C.W. (2016). P2Y12 receptor inhibition and effect of morphine in patients undergoing primary PCI for ST-segment elevation myocardial infarction. The PRIVATE-ATLANTIC study. Thromb. Haemost..

[20] Zwart B., Yazdani M., Ow K.W., Richardson J.D., Iqbal J., Gunn J.P., Storey R.F. (2020). Use of glycoprotein IIb/IIIa antagonists to prevent stent thrombosis in morphine-treated patients with ST-elevation myocardial infarction. Platelets.

[21] Roule V., Agueznai M., Sabatier R., Blanchart K., Lemaître A., Ardouin P., Collet J.-P., Milliez P., Montalescot G., Beygui F. (2017). Safety and efficacy of IIb/IIIa inhibitors in combination with highly active oral antiplatelet regimens in acute coronary syndromes: A meta-analysis of pivotal trials. Platelets.

[22] Storey R.F., Sinha A. (2016). Cangrelor for the management and prevention of arterial thrombosis. Expert Rev. Cardiovasc. Ther..

[23] Storey R.F., Bliden K.P., Ecob R., Karunakaran A., Butler K., Wei C., Tantry U., Gurbel P.A. (2011). Earlier recovery of platelet function after discontinuation of treatment with ticagrelor compared with clopidogrel in patients with high antiplatelet responses. J. Thromb. Haemost..

[24] Storey R.F., Oldroyd K.G., Wilcox R.G. (2001). Open multicentre study of the P2T receptor antagonist AR-C69931MX assessing safety, tolerability and activity in patients with acute coronary syndromes. Thromb. Haemost..

[25] Akers W.S., Oh J.J., Oestreich J.H., Ferraris S., Wethington M., Steinhubl S.R. (2010). Pharmacokinetics and pharmacodynamics of a bolus and infusion of cangrelor: A direct, parenteral P2Y12 receptor antagonist. J Clin Pharm..

[26] Harrington R.A., Stone G.W., McNulty S., White H.D., Lincoff A.M., Gibson C.M., Pollack C.V., Montalescot G., Mahaffey K.W., Kleiman N.S. (2009). Platelet inhibition with cangrelor in patients undergoing PCI. N. Engl. J. Med..

[27] Bhatt D.L., Lincoff A.M., Gibson C.M., Stone G.W., McNulty S., Montalescot G., Kleiman N.S., Goodman S.G., White H.D., Mahaffey K.W. (2009). Intravenous platelet blockade with cangrelor during PCI. N. Engl. J. Med..

[28] Bhatt D.L., Stone G.W., Mahaffey K.W., Gibson C.M., Steg P.G., Hamm C.W., Price M.J., Leonardi S., Gallup D., Bramucci E. (2013). Effect of platelet inhibition with cangrelor during PCI on ischemic events. N. Engl. J. Med..

[29] Steg P.G., Bhatt D.L., Hamm C.W., Stone G.W., Gibson C.M., Mahaffey K.W., Leonardi S., Liu T., Skerjanec S., Day J.R. (2013). Effect of cangrelor on periprocedural outcomes in percutaneous coronary interventions: A pooled analysis of patient-level data. Lancet.

[30] Alexopoulos D., Pappas C., Sfantou D., Lekakis J. (2018). Cangrelor in Percutaneous Coronary Intervention: Current Status and Perspectives. J. Cardiovasc. Pharmacol. Ther..

[31] Angiolillo D.J., Rollini F., Storey R.F., Bhatt D.L., James S., Schneider D.J., Sibbing D., So D.Y.F., Trenk D., Alexopoulos D. (2017). International Expert Consensus on Switching Platelet P2Y12 Receptor-Inhibiting Therapies. Circulation.

[32] Qamar A., Bhatt D.L. (2017). Optimizing the Use of Cangrelor in the Real World. Am. J. Cardiovasc. Drugs.

[33] Gorog D.A., Price S., Sibbing D., Baumbach A., Capodanno D., Gigante B., Halvorsen S., Huber K., Lettino M., Leonardi S. (2020). Antithrombotic therapy in patients with acute coronary syndrome complicated by cardiogenic shock or out-of-hospital cardiac arrest: A Joint Position Paper from the European Society of Cardiology (ESC) Working Group on Thrombosis, in association with the Acute Cardiovascular Care Association (ACCA) and European Association of Percutaneous Cardiovascular Interventions (EAPCI). Eur. Heart J. Cardiovasc. Pharm..

[34] Parker W.A.E., Storey R.F. (2020). Novel approaches to P2Y12 inhibition and aspirin dosing. Platelets.

[35] Parker W.A.E., Storey R.F. (2020). Pharmacology and potential role of selatogrel, a subcutaneous platelet P2Y12 receptor antagonist. Expert Opin. Emerg. Drugs.

[36] Caroff E., Meyer E., Treiber A., Hilpert K., Riederer M.A. (2014). Optimization of 2-phenyl-pyrimidine-4-carboxamides towards potent, orally bioavailable and selective P2Y(12) antagonists for inhibition of platelet aggregation. Bioorg. Med. Chem. Lett..

[37] Rey M., Kramberg M., Hess P., Morrison K., Ernst R., Haag F., Weber E., Clozel M., Baumann M., Caroff E. (2017). The reversible P2Y12 antagonist ACT-246475 causes significantly less blood loss than ticagrelor at equivalent antithrombotic efficacy in rat. Pharm. Res. Perspect..

[38] Baldoni D., Bruderer S., Krause A., Gutierrez M., Gueret P., Astruc B., Dingemanse J. (2014). A new reversible and potent P2Y12 receptor antagonist (ACT-246475): Tolerability, pharmacokinetics, and pharmacodynamics in a first-in-man trial. Clin. Drug Investig..

[39] Juif P.-E., Boehler M., Dobrow M., Ufer M., Dingemanse J. (2019). Clinical Pharmacology of the Reversible and Potent P2Y12 Receptor Antagonist ACT-246475 After Single Subcutaneous Administration in Healthy Male Subjects. J. Clin. Pharm..

[40] Ufer M., Huynh C., van Lier J.J., Caroff E., Fischer H., Dingemanse J. (2020). Absorption, distribution, metabolism and excretion of the P2Y12 receptor antagonist selatogrel after subcutaneous administration in healthy subjects. Xenobiotica.

[41] Storey R.F., Gurbel P.A., Ten Berg J., Bernaud C., Dangas G.D., Frenoux J.-M., Gorog D.A., Hmissi A., Kunadian V., James S.K. (2019). Pharmacodynamics, pharmacokinetics, and safety of single-dose subcutaneous administration of selatogrel, a novel P2Y12 receptor antagonist, in patients with chronic coronary syndromes. Eur. Heart J..

[42] Sinnaeve P.R., Fahrni G., Schelfaut D., Spirito A.S., Mueller C.H., Frenoux J.-M., Hmissi A., Bernaud C., Moccetti T., Atar S.A. (2019). Inhibition of platelet aggregation after subcutaneous administration of a single-dose of selatogrel, a novel P2Y12 antagonist, in acute myocardial infarction: A randomised open-label phase 2 study. Eur Heart J..

[43] Durrant T.N., Hers I. (2020). PI3K inhibitors in thrombosis and cardiovascular disease. Clin. Transl. Med..

[44] Garcia A., Kim S., Bhavaraju K., Schoenwaelder S.M., Kunapuli S.P. (2010). Role of phosphoinositide 3-kinase beta in platelet aggregation and thromboxane A2 generation mediated by Gi signalling pathways. Biochem. J..

[45] Sturgeon S.A., Jones C., Angus J.A., Wright C.E. (2008). Advantages of a selective beta-isoform phosphoinositide 3-kinase antagonist, an anti-thrombotic agent devoid of other cardiovascular actions in the rat. Eur. J. Pharmacol..

[46] Nylander S., Kull B., Björkman J.A., Ulvinge J.C., Oakes N., Emanuelsson B.M., Andersson M., Skärby T., Inghardt T., Fjellström O. (2012). Human target validation of phosphoinositide 3-kinase (PI3K)β: Effects on platelets and insulin sensitivity, using AZD6482 a novel PI3Kβ inhibitor. J. Thromb. Haemost..

[47] Mateo J., Ganji G., Lemech C., Burris H.A., Han S.-W., Swales K., Decordova S., DeYoung M.P., Smith D.A., Kalyana-Sundaram S. (2017). A First-Time-in-Human Study of GSK2636771, a Phosphoinositide 3 Kinase Beta-Selective Inhibitor, in Patients with Advanced Solid Tumors. Clin. Cancer Res..

[48] Parker W.A.E., Storey R.F. (2018). ‘Thrombotic Response’ in ESC Textbook of Cardiovascular Medicine.

[49] Storey R.F., Wilcox R.G., Heptinstall S. (1998). Differential effects of glycoprotein IIb/IIIa antagonists on platelet microaggregate and macroaggregate formation and effect of anticoagulant on antagonist potency. Implications for assay methodology and comparison of different antagonists. Circulation.

[50] Durrant T.N., van den Bosch M.T., Hers I. (2017). Integrin αIIbβ3 outside-in signaling. Blood.

[51] Shen B., Zhao X., O’Brien K.A., Stojanovic-Terpo A., Delaney M.K., Kim K., Cho J., Lam S.C.-T., Du X. (2013). A directional switch of integrin signalling and a new anti-thrombotic strategy. Nature.

[52] Schwarz M., Meade G., Stoll P., Ylanne J., Bassler N., Chen Y.C., Hagemeyer C.E., Ahrens I., Moran N., Kenny D. (2006). Conformation-specific blockade of the integrin GPIIb/IIIa: A novel antiplatelet strategy that selectively targets activated platelets. Circ. Res..

[53] Hohmann J.D., Wang X., Krajewski S., Selan C., Haller C.A., Straub A., Chaikof E.L., Nandurkar H.H., Hagemeyer C.E., Peter K. (2013). Delayed targeting of CD39 to activated platelet GPIIb/IIIa via a single-chain antibody: Breaking the link between antithrombotic potency and bleeding?. Blood.

[54] Sarratt K.L., Chen H., Zutter M.M., Santoro S.A., Hammer D.A., Kahn M.L. (2005). GPVI and alpha2beta1 play independent critical roles during platelet adhesion and aggregate formation to collagen under flow. Blood.

[55] Ungerer M., Rosport K., Bültmann A., Piechatzek R., Uhland K., Schlieper P., Gawaz M., Münch G. (2011). Novel antiplatelet drug revacept (Dimeric Glycoprotein VI-Fc) specifically and efficiently inhibited collagen-induced platelet aggregation without affecting general hemostasis in humans. Circulation.

[56] Schüpke S., Hein-Rothweiler R., Mayer K., Janisch M., Sibbing D., Ndrepepa G., Hilz R., Laugwitz K.-L., Bernlochner I., Gschwendtner S. (2019). Revacept, a Novel Inhibitor of Platelet Adhesion, in Patients Undergoing Elective PCI-Design and Rationale of the Randomized ISAR-PLASTER Trial. Thromb. Haemost..

[57] Lebozec K., Jandrot-Perrus M., Avenard G., Favre-Bulle O., Billiald P. (2017). Design, development and characterization of ACT017, a humanized Fab that blocks platelet’s glycoprotein VI function without causing bleeding risks. MAbs.

[58] Voors-Pette C., Lebozec K., Dogterom P., Jullien L., Billiald P., Ferlan P., Renaud L., Favre-Bulle O., Avenard G., Machacek M. (2019). Safety and Tolerability, Pharmacokinetics, and Pharmacodynamics of ACT017, an Antiplatelet GPVI (Glycoprotein VI) Fab. Arterioscler. Thromb. Vasc. Biol..

[59] O’Neill S., Robinson A., Deering A., Ryan M., Fitzgerald D.J., Moran N. (2000). The platelet integrin alpha IIbbeta 3 has an endogenous thiol isomerase activity. J. Biol. Chem..

[60] Stopa J.D., Neuberg D., Puligandla M., Furie B., Flaumenhaft R., Zwicker J.I. (2017). Protein disulfide isomerase inhibition blocks thrombin generation in humans by interfering with platelet factor V activation. JCI Insight..

[61] Zwicker J.I., Schlechter B.L., Stopa J.D., Liebman H.A., Aggarwal A., Puligandla M., Caughey T., Bauer K.A., Kuemmerle N., Wong E. (2019). Targeting protein disulfide isomerase with the flavonoid isoquercetin to improve hypercoagulability in advanced cancer. JCI Insight.

[62] Kung P.-H., Hsieh P.-W., Lin Y.-T., Lee J.-H., Chen I.-H., Wu C.-C. (2017). HPW-RX40 prevents human platelet activation by attenuating cell surface protein disulfide isomerases. Redox Biol..

[63] Judge H.M., Jennings L.K., Moliterno D.J., Hord E., Ecob R., Tricoci P., Rorick T., Kotha J., Storey R.F. (2015). PAR1 antagonists inhibit thrombin-induced platelet activation whilst leaving the PAR4-mediated response intact. Platelets.

[64] Tricoci P., Huang Z., Held C., Moliterno D.J., Armstrong P.W., Van de Werf F., White H.D., Aylward P.E., Wallentin L., Chen E. (2012). Thrombin-receptor antagonist vorapaxar in acute coronary syndromes. N. Engl. J. Med..

[65] Morrow D.A., Braunwald E., Bonaca M.P., Ameriso S.F., Dalby A.J., Fish M.P., Fox K.A.A., Lipka L.J., Liu X., Nicolau J.C. (2012). Vorapaxar in the secondary prevention of atherothrombotic events. N. Engl. J. Med..

[66] Aisiku O., Peters C.G., De Ceunynck K., Ghosh C.C., Dilks J.R., Fustolo-Gunnink S.F., Huang M., Dockendorff C., Parikh S.M., Flaumenhaft R. (2015). Parmodulins inhibit thrombus formation without inducing endothelial injury caused by vorapaxar. Blood.

[67] Wong P.C., Seiffert D., Bird J.E., Watson C.A., Bostwick J.S., Giancarli M., Allegretto N., Hua J., Harden D., Guay J. (2017). Blockade of protease-activated receptor-4 (PAR4) provides robust antithrombotic activity with low bleeding. Sci. Transl. Med..

[68] Wilson S.J., Ismat F.A., Wang Z., Cerra M., Narayan H., Raftis J., Gray T.J., Connell S., Garonzik S., Ma X. (2018). PAR4 (Protease-Activated Receptor 4) Antagonism With BMS-986120 Inhibits Human Ex Vivo Thrombus Formation. Arterioscler. Thromb. Vasc. Biol..

[69] Sargentini-Maier M.L., De Decker P., Tersteeg C., Canvin J., Callewaert F., De Winter H. (2019). Clinical pharmacology of caplacizumab for the treatment of patients with acquired thrombotic thrombocytopenic purpura. Expert Rev. Clin. Pharm..

[70] Scully M., Cataland S.R., Peyvandi F., Coppo P., Knöbl P., Kremer Hovinga J.A., Metjian A., de la Rubia J., Pavenski K., Callewaert F. (2019). Caplacizumab Treatment for Acquired Thrombotic Thrombocytopenic Purpura. N. Engl. J. Med..

[71] Libby P. (2013). Mechanisms of acute coronary syndromes and their implications for therapy. N. Engl. J. Med..

[72] Yusuf S., Mehta S.R., Chrolavicius S., Afzal R., Pogue J., Granger C.B., Budaj A., Peters R.J.G., Bassand J.-P. (2006). Fifth Organization to Assess Strategies in Acute Ischemic Syndromes Investigators. Comparison of fondaparinux and enoxaparin in acute coronary syndromes. N. Engl. J. Med..

[73] Rao S.V., Ohman E.M. (2010). Anticoagulant therapy for percutaneous coronary intervention. Circ. Cardiovasc. Interv..

[74] Montalescot G., Cohen M., Salette G., Desmet W.J., Macaya C., Aylward P.E.G., Steg P.G., White H.D., Gallo R., Steinhubl S.R. (2008). Impact of anticoagulation levels on outcomes in patients undergoing elective percutaneous coronary intervention: Insights from the STEEPLE trial. Eur. Heart J..

[75] Sumaya W., Parker W.A.E., Fretwell R., Hall I.R., Barmby D.S., Richardson J.D., Iqbal J., Adam Z., Morgan K.P., Gunn J.P. (2018). Pharmacodynamic Effects of a 6-Hour Regimen of Enoxaparin in Patients Undergoing Primary Percutaneous Coronary Intervention (PENNY PCI Study). Thromb. Haemost..

[76] Montalescot G., Zeymer U., Silvain J., Boulanger B., Cohen M., Goldstein P., Ecollan P., Combes X., Huber K., Pollack C. (2011). Intravenous enoxaparin or unfractionated heparin in primary percutaneous coronary intervention for ST-elevation myocardial infarction: The international randomised open-label ATOLL trial. Lancet.

[77] Dewilde W.J.M., Oirbans T., Verheugt F.W.A., Kelder J.C., De Smet B.J.G.L., Herrman J.-P., Adriaenssens T., Vrolix M., Heestermans A.A.C.M., Vis M.M. (2013). Use of clopidogrel with or without aspirin in patients taking oral anticoagulant therapy and undergoing percutaneous coronary intervention: An open-label, randomised, controlled trial. Lancet.

[78] Lamberts M., Gislason G.H., Olesen J.B., Kristensen S.L., Schjerning Olsen A.-M., Mikkelsen A., Christensen C.B., Lip G.Y.H., Køber L., Torp-Pedersen C. (2013). Oral anticoagulation and antiplatelets in atrial fibrillation patients after myocardial infarction and coronary intervention. J. Am. Coll. Cardiol..

[79] Oldgren J., Steg P.G., Hohnloser S.H., Lip G.Y.H., Kimura T., Nordaby M., Brueckmann M., Kleine E., Ten Berg J.M., Bhatt D.L. (2019). Dabigatran dual therapy with ticagrelor or clopidogrel after percutaneous coronary intervention in atrial fibrillation patients with or without acute coronary syndrome: A subgroup analysis from the RE-DUAL PCI trial. Eur. Heart J..

[80] Alexander J.H., Lopes R.D., James S., Kilaru R., He Y., Mohan P., Bhatt D.L., Goodman S., Verheugt F.W., Flather M. (2011). Apixaban with antiplatelet therapy after acute coronary syndrome. N. Engl. J. Med..

[81] Lopes R.D., Heizer G., Aronson R., Vora A.N., Massaro T., Mehran R., Goodman S.G., Windecker S., Darius H., Li J. (2019). Antithrombotic Therapy after Acute Coronary Syndrome or PCI in Atrial Fibrillation. N. Engl. J. Med..

[82] Mega J.L., Braunwald E., Mohanavelu S., Burton P., Poulter R., Misselwitz F., Hricak V., Barnathan E.S., Bordes P., Witkowski A. (2009). Rivaroxaban versus placebo in patients with acute coronary syndromes (ATLAS ACS-TIMI 46): A randomised, double-blind, phase II trial. Lancet.

[83] Mega J.L., Braunwald E., Wiviott S.D., Bassand J.-P., Bhatt D.L., Bode C., Burton P., Cohen M., Cook-Bruns N., Fox K.A.A. (2012). Rivaroxaban in patients with a recent acute coronary syndrome. N. Engl. J. Med..

[84] Steg P.G., Mehta S.R., Jukema J.W., Lip G.Y.H., Gibson C.M., Kovar F., Kala P., Garcia-Hernandez A., Renfurm R.W., Granger C.B. (2011). RUBY-1: A randomized, double-blind, placebo-controlled trial of the safety and tolerability of the novel oral factor Xa inhibitor darexaban (YM150) following acute coronary syndrome. Eur. Heart J..

[85] Goldstein S., Bates E.R., Bhatt D.L., Cao C., Holmes D., Kupfer S., Martinez F., Spaeder J., Weitz J.I., Ye Z. (2014). Phase 2 study of TAK-442, an oral factor Xa inhibitor, in patients following acute coronary syndrome. Thromb. Haemost..

[86] Schmaier A.H., Emsley J., Feener E.P., Gailani D., Govers-Riemslag J.W.P., Kaplan A.P., Maas C., Morrissey J.H., Renné T., Sidelmann J.J. (2019). Nomenclature of factor XI and the contact system. J. Thromb. Haemost..

[87] Puy C., Rigg R.A., McCarty O.J.T. (2016). The hemostatic role of factor XI. Thromb. Res..

[88] Renné T., Pozgajová M., Grüner S., Schuh K., Pauer H.-U., Burfeind P., Gailani D., Nieswandt B. (2005). Defective thrombus formation in mice lacking coagulation factor XII. J. Exp. Med..

[89] Renné T., Oschatz C., Seifert S., Müller F., Antovic J., Karlman M., Benz P.M. (2009). Factor XI deficiency in animal models. J. Thromb. Haemost..

[90] Chan N.C., Weitz J.I. (2019). Antithrombotic Agents. Circ. Res..

[91] Buyue Y., Whinna H.C., Sheehan J.P. (2008). The heparin-binding exosite of factor IXa is a critical regulator of plasma thrombin generation and venous thrombosis. Blood.

[92] Woodruff B., Sullenger B., Becker R.C. (2010). Antithrombotic therapy in acute coronary syndrome: How far up the coagulation cascade will we go?. Curr. Cardiol. Rep..

[93] Colman R.W. (2006). Are hemostasis and thrombosis two sides of the same coin?. J. Exp. Med..

[94] DeLoughery E.P., Olson S.R., Puy C., McCarty O.J.T., Shatzel J.J. (2019). The Safety and Efficacy of Novel Agents Targeting Factors XI and XII in Early Phase Human Trials. Semin. Thromb. Hemost..

[95] Rai V., Balters M.W., Agrawal D.K. (2019). Factors IX, XI, and XII: Potential therapeutic targets for anticoagulant therapy in atherothrombosis. Rev. Cardiovasc. Med..

[96] Al-Horani R.A. (2020). Factor XI(a) inhibitors for thrombosis: An updated patent review (2016-present). Expert Opin. Ther. Pat..

